# Variable Effects of Dispersal on Productivity of Bacterial Communities Due to Changes in Functional Trait Composition

**DOI:** 10.1371/journal.pone.0080825

**Published:** 2013-12-04

**Authors:** Ina Severin, Örjan Östman, Eva S. Lindström

**Affiliations:** 1 Department of Ecology and Genetics/Limnology, Uppsala University, Uppsala, Sweden; 2 Department of Ecology and Genetics/Animal Ecology, Uppsala University, Uppsala, Sweden; Wageningen University, The Netherlands

## Abstract

Previous studies have shown variable relationships between dispersal rate and ecosystem functioning, but the reasons for and mechanisms behind variable dispersal rate – functioning patterns are currently unknown. In this study we used six bacterial lake water communities in a laboratory experiment in order to investigate how dispersal among communities influences community productivity by evaluating three different mechanisms: 1) changes in taxonomic diversity, 2) changes in phylogenetic diversity or 3) changes in the composition of functional traits. The experiment was conducted in two phases; (A) a dialysis bag experiment where the dispersal rate among six communities was manipulated and the subsequent change in bacterial diversity and growth rate was recorded, and (B) a regrowth experiment where we manipulated available resources to study how well a taxon grows on certain organic carbon resources, i.e. their functional traits. From experiment (B) we could thus estimate changes in functional traits in communities in experiment (A). Bacterial production was affected by dispersal, but not consistently among lakes. Neither change in taxonomic or phylogenetic diversity with dispersal could explain the observed dispersal – productivity relationships. Instead, changes in trait composition with dispersal, especially the communities’ ability to use p-coumaric acid, an aromatic compound, could explain the observed dispersal – productivity relationships. Changes in this trait caused by dispersal seemed especially important for bacterial productivity in waters with a high aromaticity of the organic matter pool. We conclude that the effect of dispersal on bacterial communities can affect ecosystem functioning in different ways, through changes in functional key-traits which are important for the local environment.

## Introduction

For many types of communities there is a relationship between diversity and ecosystem functioning, i.e. a change in biodiversity may alter local ecosystem functioning [1], [2]. One concern is that habitat fragmentation affecting dispersal rates among communities may impact community functioning since dispersal among communities may change local community composition and diversity, e.g., [3]. In artificial communities productivity and diversity have been shown to peak at intermediate dispersal rates [4], [5], but other patterns have also been found [6]. The number of studies investigating the role of dispersal among communities for community functioning is, however, low and more experimental work in different types of communities is needed for general conclusions and in order to pinpoint the mechanisms behind such relationships.

A hump shaped relationship between dispersal rate and functioning has been explained by the complementarity effect, i.e. that an initial increase in dispersal adds taxa and thereby functions that contribute to community productivity, while at the highest dispersal rates, richness decreases due to regional homogenization and the consequent loss of functions causes a decrease in productivity [4], [5]. There are, thus, two assumptions to be fulfilled for the hump-shaped relationship between dispersal rate and functioning: 1) community richness is highest at intermediate dispersal rate, according to a theoretical model [3] and 2) function is positively monotonically related to richness. Previous studies indeed showed experimentally that community richness may follow dispersal rates in the expected way [4], [5]. However, there are several scenarios for non-monotonic relationships between richness and function which would lead to differing effects of dispersal. If, for example, increased richness should lead to increased function due to the complementarity effect not only the number of taxa needs to increase but rather the number of different functions, and a better measure of functional diversity may therefore be phylogenetic diversity (PD), e.g., [7], [8], since distantly related taxa are more likely to possess different functions [9]. However, PD can change independently of richness and vice versa [10], and increased richness without increasing PD may even result in reduced functioning due to increased antagonistic interactions [11], obscuring the relationship between richness and function. Thus, if PD is more important than richness for function, various outcomes of dispersal on functioning seem plausible. Finally, instead of the diversity of functional traits, the presence or abundance of some key functional traits may disproportionally affect ecosystem functioning [1], [12]. Different functional traits may be added or diluted as a consequence of dispersal; however, there is no obvious relationship between traits of importance for community functioning and dispersal, which could result in basically any relationship between dispersal rate and functioning.

Our aim was to study the effect of different dispersal rates on productivity (our ecosystem function of interest) of bacterial communities and to test alternative explanations for the patterns in function. For that purpose, we used a microscale set-up with natural bacterial lake water communities. The first hypothesis we tested was that community richness is highest at intermediate dispersal and that function (productivity) shows a hump-shaped relationship to dispersal rate due to a positive monotonic relationship between richness and function. Our second hypothesis is that PD is more important for function than richness, and since richness and PD are not necessarily related, relationships other than hump-shaped are possible between dispersal rate and function, but PD should always scale positively with productivity. The third hypothesis was that the abundance of certain functional key-traits is more important for community function than richness and PD, and that changes in trait composition following a dispersal event affect productivity. The functional trait chosen in this study was the ability of the bacteria to grow on different carbon substrates since that differs among bacteria [13], [14] and because carbon processing is central for the role of bacteria in ecosystems, e.g., [15]. Since the chemical composition of the organic matter pool in, for instance, lakes differs over space and time [16], [17], it is likely that the traits being of importance for functioning differ among environments.

The microcosm experiment was conducted in the laboratory in two parts using bacteria from six contrasting environments. In the first part (A) we induced dispersal among communities by pipetting. In the second part (B) we experimentally defined traits for the taxa in those communities by exposing them to eight different carbon substrates. Thereafter we analyzed the effect of changed dispersal rate for community growth rate as well as richness and PD in the communities in experiment (A). Finally, we evaluated the role of richness, PD and trait composition for the dispersal-function relationship found in experiment (A).

## Materials and Methods

### Ethics Statement

No permits were required to sample any of the lakes in this study. The authors also confirm that the sampling did not affect endangered or protected species.

### Sampling and Water Chemistry

Water from six lakes in central Sweden (provinces of Uppland and Västmanland) was sampled in March 2011. In order to ensure the general validity of the results, lakes that differ more than one order of magnitude in inorganic nutrient content, organic carbon content and hydroxide ions (pH) were chosen. Non-purgeable organic carbon (NPOC) was determined by measuring organic carbon after acidification and with HCl (TOC-5000, Shimadzu, Kyoto, Japan). Total nitrogen (TN) was measured spectrophotometrically (Hitachi U-2000, Hitachi, Ltd., Tokyo, Japan) as nitrate after oxidation at high temperature. Total phosphorus (TP) was also measured spectrophotometrically after oxidative hydrolysis of organically bound phosphorus. Absorbance was measured from 200 to 600 nm (1 nm intervals) using a Lambda 40 UV/VIS Spectrometer (PerkinElmer, Inc., Waltham, MA, USA). SUVA (specific UV absorbance) is the ratio of absorbance at 254 nm over total organic carbon concentration. Cell abundances were determined flow-cytometrically (CyFlow space, Partec GmbH, Münster, Germany) [18]. In short, water samples were fixed with filtered formaldehyde (3.7% final concentration) and stored at 4°C for a maximum of two days. The cells were stained and enumerated with SYTO 13 (Invitrogen, Life Technologies Ltd, Paisley, UK). Physico-chemical characteristics and cell abundances are listed in Table S1.

### Preparation of Medium and Experimental Set-up

All lake water was filtered through a 1 µm glass fiber filter (Gelman A/E, Pall Life Sciences, Port Washington, NY, USA) to remove organisms larger than bacteria. For the first experiment (hereafter termed ‘dispersal experiment’), this water was used as medium and inoculum. For the second experiment (hereafter termed ‘resource addition experiment’), this water was used as an inoculum whereas a second filtration through 0.2 µm filters (Supor®-200 Membrane Disc Filters, 47 mm; Pall Corporation, East Hills, NY, USA), two autoclaving steps and readjustment of the pH were carried out to prepare the sterile medium for the experiment.

### Dispersal Experiment

The setup is similar to the one used by Lindström and Östman [6]. From each lake one 10 L bucket was filled with the 1 µm-filtered lake water. In each bucket thirteen 10 ml dialysis bags were placed (Float-A-Lyzer® G2, 50 000 Daltons, Spectrum Europe B.V., Breda, The Netherlands), containing the natural bacterial community from that lake (triplicates for each of the four dispersal treatments) and one negative control (autoclaved MQ). The water in the buckets was mixed by vigorous air bubbling. The dialysis bags were pre-wetted in MQ water for two days before the initiation of the experiment. Three times a day bacteria were dispersed between the communities in different lake water but within each dispersal rate treatment by pipetting. At each dispersal event a metacommunity was constructed for each dispersal rate; equal volumes were taken out from all dialysis bags within a dispersal rate treatment (n = 18), mixed and kept in separate glass bottles and thereafter pipetted back into the dialysis bags. The different dispersal rate treatments were 0% (dispersal rate I), 10% (0.33 ml per dispersal event; dispersal rate II), 49% (2 ml per dispersal event; dispersal rate III), and 88% (5 ml per dispersal event; dispersal rate IV) of standing stock per day. Dispersal rate I received no dispersal during the course of the experiment, but was opened and closed to mimic the handling of the other dispersal treatments. The dialysis bags are easily opened and closed for the dispersal events via a screw cap. The experiment was run for four days at 15°C in darkness. This time period was constrained by the lifespan of the dialysis bags but still allows for sufficient population growth. At the end of the experiment samples were taken from each dialysis bag for bacterial diversity, bacterial production, abundance and absorbance. Absorbance in the original water and in dispersal treatment I and IV was measured from 200 to 600 nm (1 nm intervals). SUVA (Specific UV Absorbance) was determined at 254 nm over the total organic carbon concentration [19].

### Bacterial Production (BP)

The incorporation of ^3^H-labelled leucine into bacterial protein was determined using the modified method from Smith and Azam [20]. In short, triplicate samples and a blank (immediate addition of a final concentration of 5% TCA) were incubated in a final concentration of 100 nM ^3^H-leucine for one hour. The incubation was stopped by adding a final concentration of 5% TCA to the samples. After washing with 5% TCA and 80% Ethanol, 0.5 ml of the scintillation cocktail (Optiphase Hisafe 2, PerkinElmer, Inc., Waltham, MA, USA) were added and the samples were kept for at least 24 hours before measurement of the incorporated ^3^H-leucine (Packard Tri-Carb 2100TR Liquid Scintillation Analyzer, GMI, Inc, Ramsey, MN, USA). Results are presented as disintegrations per minute (dpm) divided by cell abundance as determined by flow cytometry (Table S1).

### Resource Addition Experiment

In order to investigate the bacterial resource use in the communities, a second experiment using the same lake water as for the dispersal experiment was set up. Volumes of 30 ml of the 1 µm-filtered water from each lake served as the inoculum which was added to 270 ml of the sterile medium (including the substrates) in order to allow re-growth of the bacterial community. A control treatment (no substrate addition) and eight duplicate carbon substrates additions resulted in 17 incubations per lake. Incubations took place in the dark at 15°C until stationary growth phase was reached (5 days). The substrates used in the experiment were an amino acid (L-arginine), a carboxylic acid (Itaconic acid), carbohydrates (D-cellobiose, N-acetyl-D-glucosamine), glycerol (lipid-backbone), polymers (α-cyclodextrin, glycogen) and an aromatic compound (p-coumaric acid). Thus the added resources varied in their chemical complexity. The carbon concentration of the added substrates was roughly 10% of the carbon content of the original lake water. Nitrogen (as NaNO_3_) and phosphorus (as Na_2_HPO_3_) were added to achieve Redfield ratios (106∶16:1 C:N:P atom ratio) of the additions. Bacterial abundance was determined at the start of the incubation and every following day of the experiment by flow cytometry (Table S1).

### Bacterial Diversity

Cells from the samples in the dispersal experiment were collected by centrifuging 3 ml of water at 17 000×G for 30 minutes in sterile eppendorff tubes. First 1.5 ml of sample was centrifuged and the supernatant discarded, and thereafter an additional 1.5 ml was added to the same tube and the procedure repeated. The cells were stored at −80°C until further processing. When bacterial growths curves reached the stationary phase (after 5 days) in the resource addition experiment, the incubation was terminated by filtration of approximately 300 ml medium onto 0.2 µm filters (Supor®-200 Membrane Disc Filters, 47 mm; Pall Corporation, East Hills, NY, USA). The filters were stored at −80°C until further processing.

For Lake 6 in the dispersal treatment, only DNA from dispersal rate I could be extracted. Similarly, no amplifiable DNA could be extracted from the negative controls (MQ water only) in the dispersal experiment, indicating low occurrence of alien bacteria from the handling of dialysis bags.

DNA from the harvested cells from both experiments was extracted using the PowerSoil® DNA Isolation kit (MO BIO Laboratories, Inc., Carlsbad, CA, USA) following the manufacturer’s instructions. DNA extracts were quality-checked on a 1% agarose gel and stored at −80°C until further processing. The bacterial hypervariable regions V3 and V4 of the 16S rRNA gene were PCR amplified and 454 pyrosequenced using forward primer 341 (5′- CCTACGGGNGGCWGCAG-3′) and the individually bar-coded reverse primer 805 (5′- GACTACHVGGGTATCTAATCC-3′) [21]. Each 20 µL PCR reaction contained 0.4 U Phusion™ high-fidelity DNA polymerase (Finnzymes, Espoo, Finland), 1X Phusion™ HF reaction buffer (Finnzymes), 200 µM of each dNTP (Life Technologies Ltd, Paisley, UK), 250 nM of each primer (Eurofins MWG, Ebersberg, Germany), 0.4 mg mL^−1^ BSA (New England Biolabs, Ipswich, UK) and 5–10 ng of extracted nucleic acid. Thermocycling was conducted with an initial denaturation step at 98°C for 30 sec, followed by 25 cycles of denaturation at 98°C for 10 sec, annealing at 50°C for 30 sec and extension at 72°C for 30 sec, and finalised with a 7-min extension step at 72°C. Three to four technical replicates were run per sample, pooled after PCR amplification and quality-checked on a 1% agarose gel. Purification was carried out using the AMPure XP purification kit (Beckman Coulter Inc., Brea, CA, USA). Nucleic acid yields were then checked on a fluorescence microplate reader (Ultra 384; Tecan Group Ltd., Männedorf, Switzerland) applying the Quant-iT PicoGreen dsDNA quantification kit (Invitrogen, Life Technologies Ltd, Paisley, UK). Finally, PCR amplicons were combined equimolarly, i.e. in equal proportions, to obtain a similar number of 454 pyrosequencing reads per sample.

The amplicon pool was 454 pyrosequenced with the AB SOLiD 4™ System (Life Technologies Corporation, Carlsbad, CA, USA) at the Uppsala Genome Center, Uppsala University (UGC; Uppsala, Sweden; http://www.igp.uu.se/Serviceverksamhet/Genomcenter/), using Titanium chemistry. Sequences were, prior to analyses, quality-checked and truncated to 400 bases. Each data set was individually processed with AmpliconNoise to reduce the number of 454 sequencing and PCR artifacts, and PCR chimeras [22]. 454 pyrosequencing reads have been deposited in the National Center for Biotechnology Information Sequence Read Archive (NCBI-SRA) under accession number SRP014765. After processing the sequencing information using AmpliconNoise an average of 4000 (1589–9161) reads per sample were obtained.

Differences in composition between communities (β-diversity) were calculated as Morisita-Horn (MH) dissimilarity index that is relatively insensitive to differences in number of sequences between samples (vegan package in R 2.13, [23], [24]). Non-metric multi-dimensional scaling (nMDS) as well as SIMPER (SIMilar PERcentage) analyses to identify OTUs mainly responsible for changes in community composition were carried out in PAST [25].

α-diversity (richness) was estimated as Chao1 [26] for operational taxonomic units (OTUs) defined by complete linkage clustering at 97% sequence similarity. Although Chao1 is less sensitive to differences in sample depth than many other α-diversity estimates, it is important to compare different communities at the same sample depth [27]. Therefore we subsampled all samples 1000 times to 1730 reads each using an in-house script in MatLab (Matlab R2012b, MathWorks Inc., Natick, MA, USA) and used the average Chao1 for each community as our estimate of local richness. This excluded two samples (one parallel of samples 1-III and 3-II each) with too few reads.

Because the number of unique sequences was many times larger than the number of variable sites in the sequence we had to build the phylogenetic tree for our measure of phylogenetic diversity from a subsample of taxa. Because we are interested in productivity it is likely the most common taxa that contribute most to the productivity we built a tree for the most common taxa, using sequences that had an average relative abundance ≥1% across all samples (zero-densities included), or an average relative abundance ≥3% in any triplicate of the same dispersal treatment in any lake, ensuring taxa abundant in one lake or treatment was also included. There were 41 taxa that fulfilled any of these criteria, constituting 57% of all sequences in the dispersal experiment, which had 291 variable sites in the sequenced 16S rRNA gene. We constructed phylogenetic trees with MrBayes 3.2 [28] using a generalized time reversible (GTR) evolutionary model with gamma-distributed rate variation across variable sites. The branch length prior was set to a uniform clock that had a better likelihood than an unconstrained branch length prior (harmonic means of likelihood −4738 and −4694, respectively). The standard deviations of split frequencies after 100 000 generations was 0.013 indicating that most nodes were well supported for these 41 taxa. We built one consensus tree from the last 100 sampled posterior probability distributions. Because many of these taxa were present in most communities there was little difference in PD based on presence and absence. Instead we calculated phylogenetic diversity as mean pairwise phylogenetic distance (MPD) between all individuals of these 41 taxa in each community from the consensus tree in the ‘picante’-package for R [29]. We used z-transformed values of MPD calculated from 1000 iterations of each community. To calculate dispersion in MPD from the phylogenetic uncertainty we calculated the z-transformed MPD values for each community from each of the 100 individual trees and calculated 95% confidence intervals. To study how trait values (resource use, r*_ij_*, see below) were distributed across the phylogenetic tree we calculated *k*-statistics [29] from the 100 single trees using the ‘picante’-package for R [29]. Low *k*-values indicate that traits are phylogenetically dispersed whereas higher values indicate traits that are more phylogenetically clustered. P-values are calculated through a randomization (here 1000 randomizations) of the observed trait values. As we used a subsample of taxa in the phylogeny we have to assume the changes in PD and *k*-values among these 41 taxa are representative for the entire community, which is motivated by the high coverage of sequences, in average 57% of all reads.

### Resource Use

The resource use width of each taxon was defined based on the changes in relative abundance of each taxon (i.e. each unique sequence) in the eight carbon source additions relative to the control (c) in the resource addition experiment. For each taxa *i* in lake *l* we calculated the relative growth rate in the control as r*_icl_* = ln(N*_icl_*/N*_i0l_*), where N*_icl_* = % of taxa *i* in the control of lake *l*, N*_i0l_* = % of taxa *i* at start of experiment in lake *l*. Population growth response of taxa *i* to resource addition *j* was calculated as r*_ijl_*–r*_icl_*, where r*_ijl_* is the *j* resource specific relative growth rate of taxa *i* in lake *l*, r*_ijl_* = ln(N*_ijl_*/N*_0il_*)–r*_ic l_* = ln(N*_ijl_*/N*_0il_*)−ln(N*_icl_*/N*_0il_*)  =  ln(N*_ijl_*/N*_icl_*) where N*_ijl_* = % of taxa *i* in resource addition *j* in lake *l*. As r*_ijl_* cannot handle zero values of N*_ijl_* or N*_icl_* we only calculated r*_ijl_* for N*_ijl_* and N*_icl_*>0. r*_ijl_* was first averaged replicates and then over all lakes to calculate taxon specific (*i*) response to each resource (*j*) addition, r*_ij_*.

We matched the taxa from the resource experiment with the taxa from the dispersal treatment, based on 100% agreement of the 16S rRNA gene sequence. In total 831 taxa overlapped between the two experiments (83% of all sequences in the dispersal treatment and 79% of all the sequences in the resource experiment). Thus, the taxonomical overlap between the two experiments was high enough for the results from the resource experiment to be relevant for the dispersal experiment. From these 831 taxa we calculated the community average ability to grow on resource *j* for each community (*C*) in the dispersal treatment, *R_jC_*, as the average growth rates (r*_ij_*) across all 831 taxa weighted for their relative abundance. Higher values indicate communities that are, on average, growing better on a resource *j*. Thus, we assume all taxa specific responses are additive and there are no interactive (antagonistic, synergistic) effects between taxa.

### Statistics

ANOVAs were run to test for the effect of lake identity and dispersal treatments on bacterial production (bulk productivity/numbers of bacterial cells), richness and MPD. To test for associations between bacterial production and the continuous explanatory variables richness and MPD we did ANCOVAs with lake identity as a class variable and interactions between lake identity and the respective covariates. To evaluate if the importance of trait composition for productivity we transformed the community specific *R_jC_* values for all eight resources into principal components. These components were then tested towards BP in ANCOVAs as above. The substrates correlated with the components explaining significant variation in BP were then tested separately using ANCOVA.

To study how community level traits depended on the aromatic content of the water, the strength of the relationship between *R_jC_* and BP, as determined by the t-value from regression analysis, was correlated to SUVA (ratio of lake water absorbance at 254 nm over total organic carbon concentration). Regression analyses were also used to determine the strength of the relationship between richness and BP as well as PD and BP.

All data are available electronically upon request.

## Results

The composition of the bacterial communities became more similar to each other with increasing dispersal (Fig. S1). The original lake water bacterial community of lake 5 was most dissimilar to all other lakes and showed the largest change in BCC due to dispersal (MH disp I–IV [Morisita-Horn dissimilarity index between dispersal rate I and IV] = 0.85) while Lake 3 showed the smallest change in BCC due to dispersal (MH disp I–IV = 0.35). Based on SIMPER analysis four OTUs contributed to more than 5% of the changes in community composition between dispersal rate I and IV in all the lakes. Two of these OTUs were most abundant (1.3–4.7% of all sequences) in the non-dispersed communities of lake 3, while the other two OTUs were most abundant (0.6–6.7% of all sequences) in the non-dispersed communities of lake 2. The former two taxa were associated with a high ability to grow on p-coumaric acid and glycerol while the latter two did not show strong growth on any of the tested resources.

**Figure 1:**
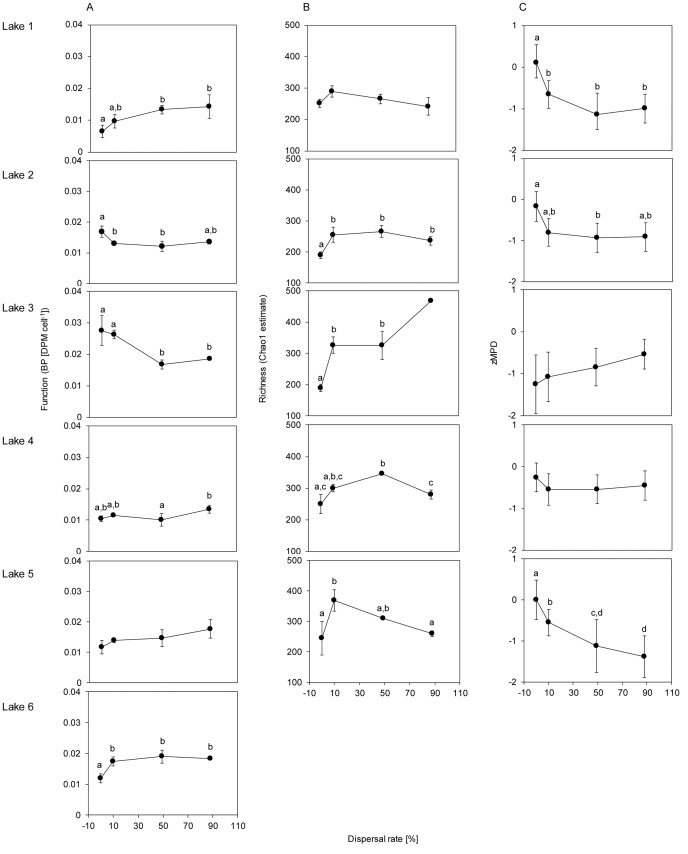
The influence of dispersal on community functioning (A), taxonomic (B) and phylogenetic diversity (C). Community functioning is assessed as per cell bacterial production (A), local richness of the bacterial community estimated by Chao1 (B), and average phylogenetic distance between bacteria in a community estimated as standardized effect size of MPD (C) for each lake (1–6) in relation to dispersal rate (I–IV). Error bars are standard errors of within treatment replicates in (A and B), and standard deviations calculated from 100 single trees in (C). No replicates were available for dispersal rate IV in lake 3 in (B). Lake 6 is missing in (B) and (C) because DNA could be extracted from dispersal rate I only. Bars with different letters denote significant differences between dispersal treatments within one lake, assessed form Tukey’s post-hoc test in one-way ANOVAs.

Dispersal had different effects on bacterial production (BP) depending on lake identity, indicated by an interaction between lake identity and dispersal (F_15,45_ = 7.46, p<0.001), i.e. Lake 2 and 3 showed decreased BP with increasing dispersal (Fig. 1A) while in the other lakes there was either a positive (Lake 1 and 6) or a close to positive (but non-significant) relationship between dispersal and BP (Lake 4 and 5, Fig. 1A). No lake showed a hump-shaped relationship with dispersal.

**Figure 2:**
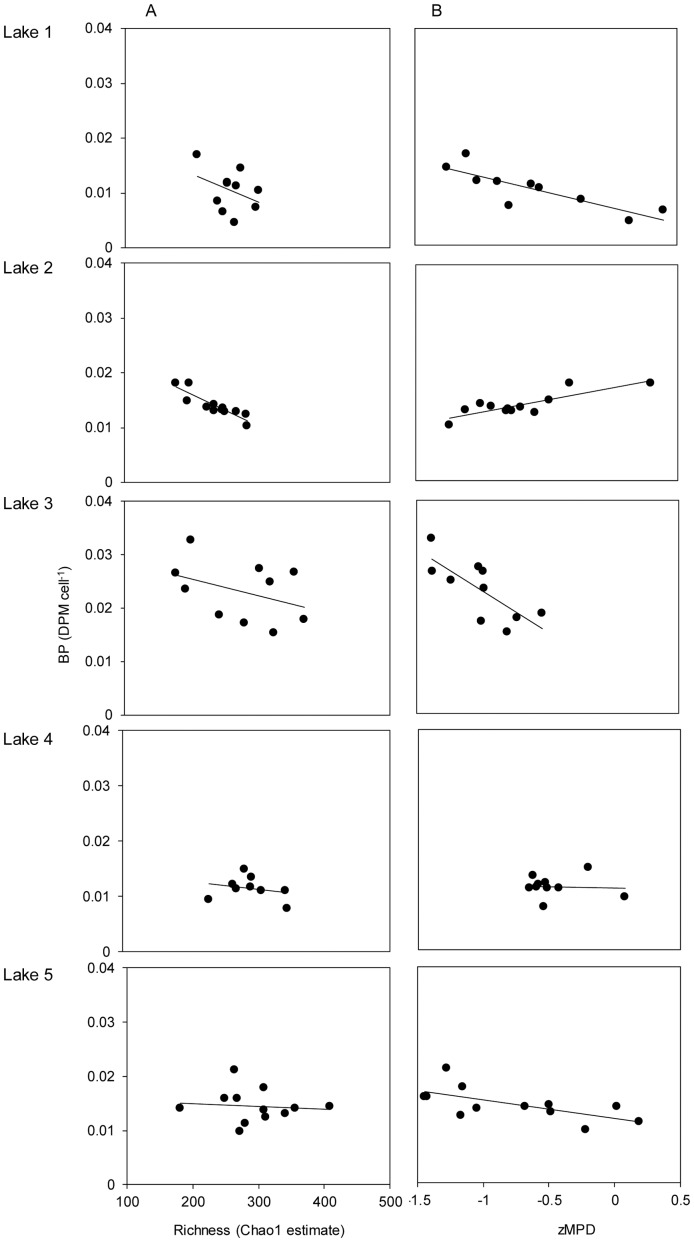
Associations between functioning and richness (A), and functioning and average phylogenetic distance (B). Community functioning is assessed as per cell bacterial production, local richness of the bacterial community estimated by Chao1 (A), and average phylogenetic distance between bacteria in a community estimated as standardized effect size of MPD (B).

Also bacterial richness (Chao1) showed a significant interaction between dispersal and lake identity (F_12,36_ = 3.1, p = 0.004), but local richness peaked at intermediate dispersal treatments in all lakes, although this was not always significant (Fig. 1B). An ANCOVA showed that, opposite to our expectations, richness was negatively associated with BP (F_1,47_ = 5.2, t_1,47_ = −2.5, p = 0.02, Fig. 2A). For Lake 6, only DNA from dispersal rate I could be extracted and, hence, data associated with the bacterial community are missing for dispersal rates II–IV.

The mean phylogenetic distance (MPD) of the consensus tree also showed an interaction between dispersal and lake identity (F_12,36_ = 7.5, p<0.001, Fig. 1C). There were different responses of BP to MPD in the different lakes, as indicated by an interaction term between lake and MPD on BP (F_4,44_ = 8.3, p<0.001). Even when accounting for the phylogenetic uncertainty, the interaction term between MPD and BP was evident, the 95% confidence of the interaction term from the 100 single trees was F = 2.6–11.9 (p<0.05). MPD was weakly negatively associated with BP in three lakes (1, 3, 5, i.e. lower BP when taxa were less related), positively associated in only one (Lake 2) and not showing a clear trend in another lake (Lake 4) (Fig. 2B).

**Figure 3:**
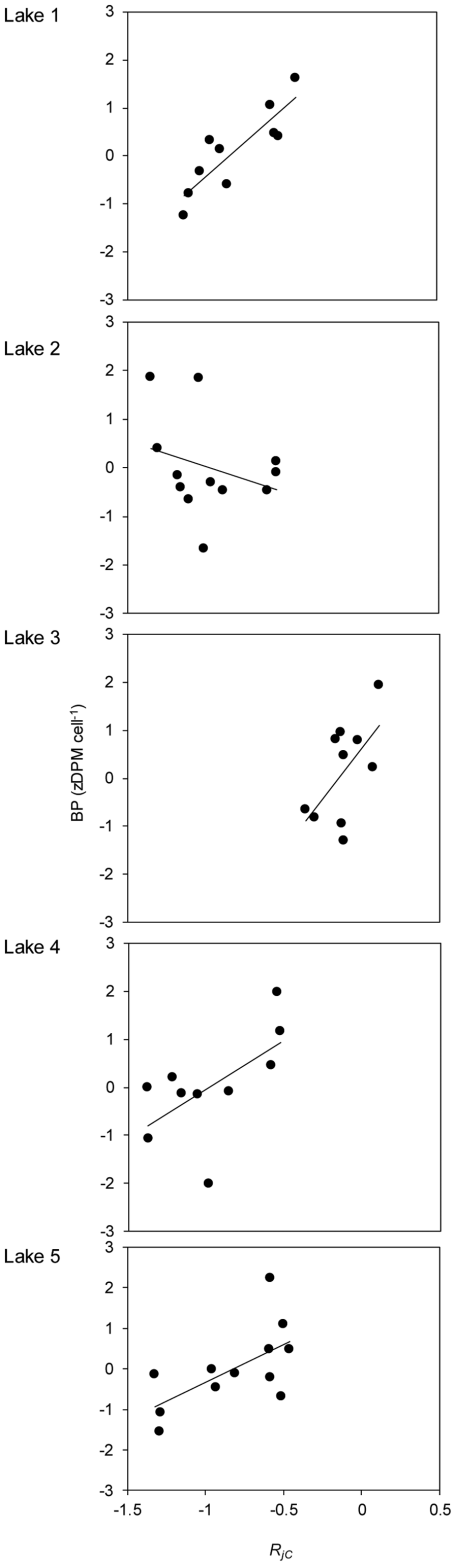
Association between functioning and the average ability of the community to grow on p-coumaric acid. Community functioning is assessed as per cell bacterial production.

The first and fourth PC axes of the trait matrix were associated with BP (ANCOVA: PC 1: F_1,47_ = 15; PC 4: F_1,47_ = 12, p<0.001). PC 1 was mainly associated with the communities’ ability to use alpha-cyclodextrin (r = 0.71), glycerol (r = 0.72) and p-coumaric acid (r = 0.83) well. The fourth PC was associated with communities’ ability to use L-arginine (r = −0.36). Of these four resource traits, variation in the communities’ ability to grow on p-coumaric acid best explained variation in BP (ANCOVA: F_1,44_ = 23, p<0.001; interaction term with lake identity: F_4,44_ = 4.6, p = 0.003, all three other resources traits with p>0.2). Hence, BP was generally strongest associated with the communities’ average ability to grow on p-coumaric acid (*R_jC_*, see Methods), but the strength differed among lakes, and was even negative in lake 2 (Fig. 3).

**Figure 4:**
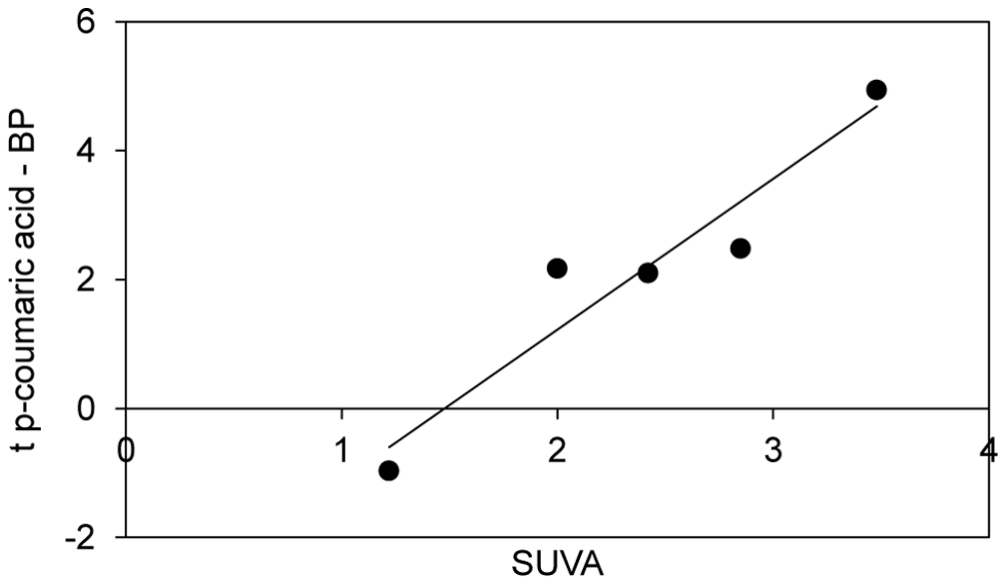
Association between SUVA and the relationship between functioning and the growth on p-coumaric acid. SUVA is the specific UV absorbance at 254

Since the importance of the functional traits for BP differed among lakes and thereby should depend on the local environment we tested if the potential ability of a community to grow on an aromatic compound (p-coumaric acid) differed in importance for BP with differing amount of aromatic compounds in the organic matter pool. To do so, we used SUVA, specific UV absorbance, which is the ratio of absorbance at 254 nm over total organic carbon concentration, and which indicates the proportion of aromatic compounds in the water [19]. SUVA was positively related to the t-value from the within lake regressions between *R_jC_* of p-coumaric acid and BP (r^2^ = 0.91, p = 0.03, Fig. 4). Thus, in lakes with a high SUVA value, the trait of growth on an aromatic compound was of great positive importance for bacterial production, while it was of less importance, and even negative, in lakes of low SUVA. The taxonomic specific ability to grow on p-coumaric acid was not clustered in the phylogeny (average k = 0.018, p>0.5). Neither was the ability to grow on any other of the carbon substrate in the experiment (average k<0.027, p>0.25 for all).

## Discussion

Our main finding is that the patterns of the relationship between dispersal and productivity differed among lakes, despite the fact that there was hump-shaped relationship between dispersal rate and richness in all lakes. Community trait composition best explained the patterns in productivity, with an especially strong role of the ability to use p-coumaric acid (an aromatic compound). In contrast, neither increased richness nor phylogenetic diversity (PD) was associated with higher productivity. Since community trait composition cannot be easily predicted from dispersal rates, variable dispersal-productivity relationships are to be expected. Further, our results indicate that the local environment is imperative for the effect the traits of the immigrating cells have on ecosystem functioning. The dispersal-productivity pattern is, thus, context-dependent.

In the lakes with a positive trend between dispersal rate and productivity (Lake 1 and Lake 5) the increase in productivity coincided with a change in community composition greatly due to an increase in abundance of two betaproteobacteria, one of them performing well on p-coumaric acid. Hence, the import of this taxon due to dispersal seems to have contributed to the increasing productivity with increasing dispersal in these lakes. This taxon was, according to sequence match in the ribosomal data base project (RDP), a betaproteobacterium with closest matches (similarity score 1) to an uncultivated *Limnohabitans* (from Lake Gossenköllersee AJ290026). It originated from Lake 3 in which dispersal instead decreased its relative abundance when other taxa (not associated with growing well on any of the tested resources) were introduced. As a consequence of the decrease of the seemingly locally well-adapted taxon the community’s ability to use p-coumaric acid decreased, and consequently productivity decreased (Fig. 3) with increasing dispersal in this lake (Fig. 1A).

In general the more aromatic the organic matter pool in the water the larger the positive effect of the community’s ability to use p-coumaric acid on productivity, i.e. the effect was greatest in the lakes which had the highest TOC concentrations and also the highest SUVA values. In the other lakes the before-mentioned taxon growing well on p-coumaric acid also increased in abundance with increasing dispersal and was disproportionally important for the change in BCC. However, these lakes were poor in aromatic compounds, and the trait to grow well these compounds appeared to be of less importance for functioning here.

While we only have data from five lakes, the general outcome of this study is nonetheless that there seems to be a link between local environmental properties and how ecosystem functions respond to variation in species composition due to trait-related changes at the community level. In our study the ability to use an aromatic compound seemed important in environments where aromatic compounds made up a great part of the organic matter pool. In general the organic matter pool in nature is poorly characterized, but the development of new methods has opened up for new possibilities [30], [31] and future studies may show which other bacterial traits are of importance for productivity in different types of environments. In lakes of lower SUVA, such as Lake 2, it can be suspected that growth on less complex organic molecules would be a more important trait, although this trait could not be identified here. The lakes sampled in our study showed SUVA values ranging from 1.22 to 3.48 and, thus, fall well within but do not span the range of SUVA observed in freshwater systems (e.g., 0.6–5.1 and 0.2–6.5 as observed by [19] and [32], respectively). Thus, one approach to identify more functional traits in future studies may be to include environments from any of the extremes in the natural range of SUVA.

Among the 41 most abundant taxa for which we constructed the phylogenetic tree, the efficiency to grow on a certain resource was not phylogenetically clustered. A recent study suggests that although bacterial traits are in general phylogenetically clustered [33], the ability to process different carbon sources, which have relatively simple genetic mechanisms, are phylogenetically dispersed compared to more complex traits such as photosynthesis and denitrification. The results may change using a larger phylogenetic tree, but we can conclude that among the most common, and hence functional important, taxa in this study, functional groups as defined by carbon processing ability cannot be easily defined based on the 16S rRNA phylogeny.

The advantage of using a bacterial model community is that more or less intact communities can be brought into the lab and easily manipulated over short temporal and spatial scales. It is important to note that results from a sequence match analysis in the Ribosomal Data Base project (RDP) showed that the taxa identified were closely related to some typical freshwater bacteria (results not shown), suggesting that the results obtained here are not artificial due to lab conditions, but represent processes that occur in nature. A disadvantage of using aquatic bacteria as model systems is, however, that the organisms are travelling with their media. Hence, a dispersal event is prone to change local environmental conditions, which also may influence bacterial function. To circumvent this problem we conducted the experiment in dialysis bags which act as a barrier for bacterial cells but allow diffusion of water and nutrients, thereby keeping the environment in the bags as close as possible to the original conditions (i.e. those in the buckets). However, a change of water chemistry due to the pipetting effort cannot be fully excluded. We observed that the differences in absorbance between the water in the dialysis bags from different lakes decreased at high dispersal compared to in the water in the buckets. However, we did not find that the change in cell-specific production and the change in absorbance in the bags receiving the highest dispersal (averaged over 200–600 nm) were correlated (r^2^ = 0.02) and we conclude that the observed patterns in production were due to the actual dispersal of cells and not due to changes in the local habitat conditions in the dialysis bags.

### Conclusions

Our experimental study showed that in bacterial communities, changes in trait composition following dispersal rather than changes in diversity *per se* have significant but varying consequences for dispersal-productivity relationships. The effect of dispersal on function is context dependent, i.e. relies on how the trait composition changes in which kind of environment. Community trait levels are attributes not easily predicted from dispersal rate, local richness or phylogenetic diversity within the community. Consequently, effects of changed dispersal rates for community functioning are not easy to predict.

## Supporting Information

Figure S1 **Results from a non-metric multi-dimensional scaling (nMDS) analysis.** Depicted is the change in bacterial community composition with increasing dispersal (ori: original lake water community, I–IV: dispersal rate I–V) in Lake 1–6. Note that for Lake 4 the original lake water community is missing and that for Lake 6 only dispersal rate I could be analyzed.
(TIF)

Table S1 **Physico-chemical and biological characteristics of the study systems.** TP: total phosphorus, TN: total nitrogen, TOC: total organic carbon, SUVA: specific UV absorbance (254 nm).
(DOCX)
