# Supplementary figures and images for: Variable Effects of Dispersal on Productivity of Bacterial Communities Due to Changes in Functional Trait Composition
Source: PLoS One. 2013 Dec 4;8(12):e80825. doi: 10.1371/journal.pone.0080825 (PMC3851979; doi:10.1371/journal.pone.0080825)

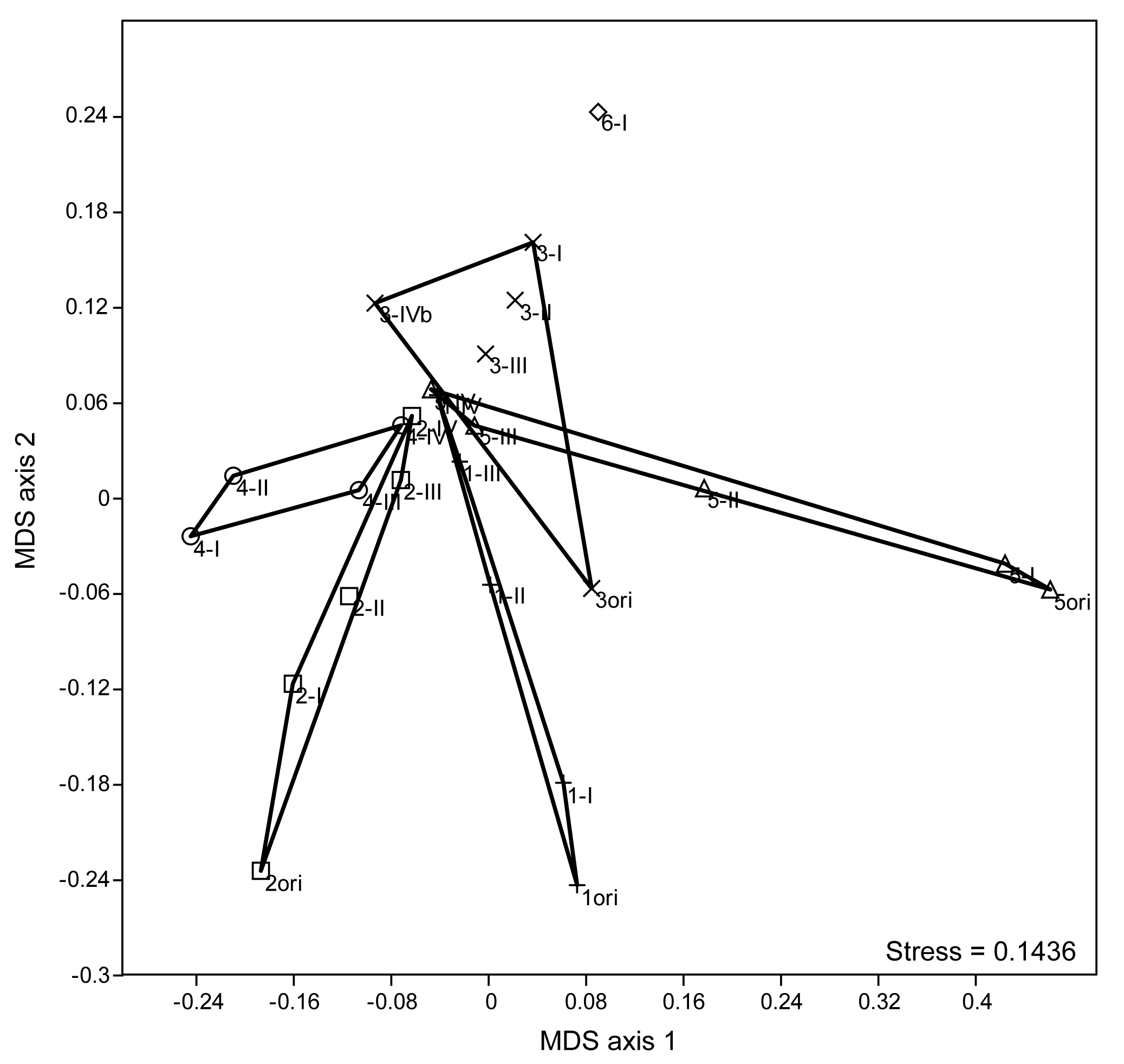

Supplement: Figure S1 — Results from a non-metric multi-dimensional scaling (nMDS) analysis. Depicted is the change in bacterial community composition with increasing dispersal (ori: original lake water community, I–IV: dispersal rate I–V) in Lake 1–6. Note that for Lake 4 the original lake water community is missing and that for Lake 6 only dispersal rate I could be analyzed. (TIF) [file pone.0080825.s001.tif]
