# Supplementary material for: Variable Effects of Dispersal on Productivity of Bacterial Communities Due to Changes in Functional Trait Composition
Source: PLoS One. 2013 Dec 4;8(12):e80825. doi: 10.1371/journal.pone.0080825 (PMC3851979; doi:10.1371/journal.pone.0080825)
Supplement: Table S1 — Physico-chemical and biological characteristics of the study systems. TP: total phosphorus, TN: total nitrogen, TOC: total organic carbon, SUVA: specific UV absorbance (254 nm). (DOCX) [file pone.0080825.s002.docx]

**Table S1. Physico-chemical and biological characteristics of the study systems.**

| Nr | Lake | pH | TP | TN | TOC | Bacterial Abundance | SUVA |
| --- | --- | --- | --- | --- | --- | --- | --- |
|  |  |  | [µg L^-1^] | [mg L^-1^] | [mg L^-1^] | [10^6^ cells mL^-1^] | (254 nm) |
| 1 | Siggeforasjön | 6.5 | 6.19 | 0.94 | 19.43 | 3.21 | 3.48 |
| 2 | Långsjön | 7.6 | 7.32 | 1.95 | 8.24 | 6.47 | 1.22 |
| 3 | Funbosjön | 7.2 | 33.02 | 3.89 | 35.89 | 7.29 | 2.42 |
| 4 | Valloxen | 7.5 | 12.57 | 3.21 | 15.77 | 3.00 | 2.20 |
| 5 | Svarttjärn | 5.6 | 3.19 | 1.02 | 28.32 | 1.77 | 2.85 |
| 6 | Ljustjärn | 6.2 | 1.31 | 0.31 | 2.15 | 2.69 | 2.31 |

TP: total phosphorus, TN: total nitrogen, TOC: total organic carbon, SUVA: specific UV absorbance (254 nm).
